# Hand Grip Strength and Its Sociodemographic and Health Correlates among Older Adult Men and Women (50 Years and Older) in Indonesia

**DOI:** 10.1155/2018/3265041

**Published:** 2018-12-03

**Authors:** Supa Pengpid, Karl Peltzer

**Affiliations:** ^1^ASEAN Institute for Health Development, Mahidol University, Salaya, Thailand; ^2^Department of Research & Innovation, University of Limpopo, Turfloop, South Africa; ^3^Department for Management of Science and Technology Development, Ton Duc Thang University, Ho Chi Minh City, Vietnam; ^4^Faculty of Pharmacy, Ton Duc Thang University, Ho Chi Minh City, Vietnam

## Abstract

**Objective:**

There is lack of knowledge about the patterns and correlates of hand grip strength (HGS) of older adults in Indonesia. This study aims to assess sociodemographic and health determinants of HGS among older adult men and women in Indonesia.

**Methods:**

Participants were 7097 individuals of 50 years and older (mean age 61.2 years, SD=9.4) that participated in the cross-sectional Indonesia Family Life Survey (IFLS-5) in 2014-15. The assessment measures included a questionnaire on sociodemographic characteristics and health variables and anthropometric and HGS measurements. Linear multivariable regression analysis was conducted to estimate the association of social and health variables and HGS.

**Results:**

The mean HGS was 28.2 kgs for men and 17.2 kgs for women. In adjusted linear regression analysis among both men and women, height, being overweight or obese, and having a good self-rated health status were positively associated with HGS, while age, having underweight, low cognitive functioning, and functional disability were negatively associated with HGS. In addition, among men, higher education and medium economic background were positive and having two or more chronic conditions, having severe depressive symptoms, and having moderate sleep impairment were negatively associated with HGS.

**Conclusion:**

The study contributed to a better understanding of patterns and correlates of HGS among older adults in Indonesia. Gender-specific and health related interventions may be needed so as to improve the physical functioning of the growing older populace in Indonesia.

## 1. Background

Hand grip strength (HGS) is used as an indicator of overall body muscle function and a proxy assessment measure of physical health, in particular among older persons [1]. Anthropometric traits (low height [2–4], underweight [3, 5], not having obesity [6]), as well as higher sitting time and lower practice of physical activity [4], are risk factors for low HGS. Various studies found that older adults with poorer self-rated health had lower HGS [5, 7–9]. Poorer cognitive functioning has been found negatively associated with HGS [3, 4, 10]. Poorer mental health status, including depressive symptoms and sleep problems, has been found to be inversely correlated with HGS among older adults in various countries [10, 11]. In the Irish Longitudinal Study on ageing among older adults, HGS was inversely associated with incident depression [12].

There is lack of knowledge about the pattern and correlates of HGS of older men and women in Indonesia. This study aims to investigate sociodemographic and health determinants of HGS among older adult men and women in national population-based survey in Indonesia.

## 2. Methods

### 2.1. Sample and Procedure

Data were analysed cross-sectionally from the “Indonesia Family Life Survey (IFLS-5)” in 2014-2015 [13]. The IFLS-5 used a multistage stratified sampling design [13]. The sampling frame of the first survey of the IFLS-1 in 1993 was based on households from 321 enumeration areas in 13 out of 27 provinces that were selected representing 83% of the Indonesian population in 1993. We followed the methods of Peltzer et al. 2018 [14]. In all, 7097 individuals 50 years and older individuals were included with complete HGS measurements; 311 were excluded from the sample “since they reported to have had any surgery, swelling, inflammation, severe pain, or injury in one or both hands in the past 6 months” [13].

The response rate was above 90%. The IFLS has been approved by ethics review boards of RAND and University of Gadjah Mada in Indonesia [13]. Written informed consent was obtained from all respondents prior to data collection.

### 2.2. Measures

#### 2.2.1. Outcome Variable

*Hand grip strength *was estimated using a “Baseline Smedley Spring type dynamometer” (calibrated daily), on “each hand twice, beginning with the dominant hand, alternating hands in between measurements” [13]. A mean HGS (kg) variable was created from all four measurements.

#### 2.2.2. Exposure Variables

*Sociodemographic factor* questions included age, sex, formal education, residential status (urban or rural), subjective socioeconomic background, and province. Subjective economic status was assessed with the question “Please imagine a six-step ladder where on the bottom (the first step), stand the poorest people, and on the highest step (the sixth step), stand the richest people. On which [economic] step are you today?” The answers ranged from (1) poorest to (6) richest [13]. Economic steps 1 to 2 were classified as poor, 3 as medium, and 4 to 6 as rich economic status. Provinces were grouped into three regions, Sumatra, Java, and major island groups.

*Anthropometric Measurements.* Heights were recorded to the nearest millimetre by using a Seca plastic height board (model 213) [13]. Weights were measured to the nearest tenth of a kilogram using a Camry model EB1003 scale [13]. Body mass index (BMI) was calculated as weight in kg divided by height in metre squared and classified according to Asian criteria: normal weight (18.5 to <23.0 kg/m^2^), overweight (23.0 to <25.0 kg/m^2^), and 25+ kg/m^2^ as obese [15].

*Cognitive functioning* was measured with items from the Telephone Survey of Cognitive Status (TICS) [16], which was administered in a face-to-face interview in this study. The TICS included awareness of the date and day of the week, and a self-reported memory question, with response options of excellent, very good, good, fair, and poor. Then the respondent was asked to serially subtract 7 from 100. Then an immediate and delayed word recall of 10 nouns was given [13]. Total scores ranged from 0 to 34, and scores of 13 or lower were considered low.

*Self-rated health status *was assessed with one item, “In general, how is your health?” (response options ranged from 1=very healthy, 2=somewhat healthy, 3=somewhat unhealthy, and 4=unhealthy) [13]. The self-rated health scores were categorized into three groups, very healthy=1, somewhat healthy=2, and somewhat unhealthy or unhealthy=3.

*Functional disability* was measured by Activities of Daily Living (ADL) (5 items) and Instrumental Activities of Daily Living (IADL) (6 items) [17, 18]. ADL questions included the extent of having difficulty in performing dressing, eating, and other activities (Cronbach alpha 0.84). Answers were categorized as follows: “have no difficulty; have difficulty but can still do it; have difficulty and need help; cannot do it”. Responses were dichotomized into 1=one or more difficulties and 0=able, no difficulty. IADL questions included the extent of having difficulty in doing household chores, such as preparing meals and shopping (Cronbach alpha 0.91). A dichotomized functional disability score was constructed and ADL/IADL disability classified as having problems with in no, one, or two or more ADL/IADL items.

*Chronic medical condition* was assessed with the question, “has a doctor/paramedic/nurse/midwife ever told you that you had…?” (“hypertension, diabetes or high blood sugar, tuberculosis, asthma, other lung conditions, heart attack, coronary heart disease, angina or other heart problems, liver, stroke, cancer of malignant tumor, arthritis or rheumatism, high cholesterol (total or LDL), kidney diseases (except for tumor or cancer), stomach or other digestive disease, emotional, nervous of psychiatric problem, and memory-related disease”) (yes, no) [13]. Responses were added up and dichotomized into having no, one, or two or more chronic conditions.

*Depression symptoms* were measured with the* Centres for Epidemiologic Studies Depression Scale (CES-D: *10 items) and scores of 15 or more were identified as having severe depressive symptoms [19] (Cronbach alpha 0.67).

*Sleep disturbance* was assessed with five items from the “Patient-Reported Outcomes Measurement Information System (PROMIS)” sleep disturbance measure [20]. A sample item was, “I had difficulty falling a asleep.” Responses ranged from 1=not at all to 5= very much (Cronbach's alpha = 0.68). Moderate sleep disturbance was classified as having a score of three to five on the averaged mean items.

*Sleep related impairment* was assessed with five items from the PROMIS sleep impairment measure [21]. A sample item was, “I had a hard time concentrating because of poor sleep.” Response options ranged from 1=not at all to 5= very much. (Cronbach's alpha = 0.82). Moderate sleep related impairment was classified as having a score of three to five on the averaged mean items.

*Physical activity* was assessed with a shortened version of the “International Physical Activity Questionnaire (IPAQ) short version, for the last 7 days (IPAQ-S7S)” [22]. Physical activity was categorized following the IPAQ scoring protocol [23] as low, moderate, and high physical activity.

## 3. Data Analysis

Descriptive statistics were computed to describe the sample and HGS. Linear multivariable regression was utilized for assessing the impact of explanatory variables on the outcome of HGS (dependent variable) for men and women, separately. Only statistically significant variables in unadjusted linear regression analyses were subsequently included in the adjusted linear regression analysis. Missing data were excluded from the analysis. All study variables that were statistically significant at the p <.05 level in bivariate analyses were subsequently included in the multivariable models. Multicollinearity between variables was measured with variance inflation factors, none of which exceeded critical values.* P* < 0.05 was considered significant. “Cross-section analysis weights were applied to correct both for sample attrition from 1993 to 2014 and then to correct for the fact that the IFLS1 sample design included oversampling in urban areas and off Java. The cross-section weights are matched to the 2014 Indonesian population, again in the 13 IFLS provinces, in order to make the attrition-adjusted IFLS sample representative of the 2014 Indonesian population in those provinces.” [13, 24]. Both the 95% confidence intervals and P values were adjusted considering the survey design of the study. All statistical procedures were done with STATA software version 13.0 (Stata Corporation, College Station, TX, USA).

## 4. Results

### 4.1. Descriptive Results

The total sample included 7097 persons 50 years or older (mean age 61.2 years, SD=9.4), 48.8% were men and 51.2% were women. More than half of the participants (54.8%) were between 50 to 59 years old, while only 3.7% were 80 years or older. A significant proportion (15.7%) had no formal education and more than half (56.4%) had elementary education. In all, 42.1% described themselves as having medium economic status, 52.1% were living in urban areas, and more than half (58.1%) lived in Java. Regarding anthropometric traits, the mean body height was 153.5 cms (159.7 cms for men and 147.8 cms for women) and 13.% of participants measured having underweight and 47.4% as general overweight or obese and 45.8% as having central obesity. A large proportion of older adults (43.9%) was physically inactive. In terms of health variables, 30.5% rated themselves as being unhealthy, 29.2% had a low cognitive functioning score, 27.8% had one or more functional disability, and 47.4% had been diagnosed with having one or more chronic conditions. Regarding mental health, 5.5% of participants reported severe depressive symptoms, 14.4% moderate sleep disturbance, and 14.0% moderate sleep impairment. The mean HGS for men was 28.2 kgs and for women 17.2 kgs (see Table 1).

### 4.2. Associations with HGS

In adjusted linear regression analysis among both men and women, height, being overweight or obese, and having a good self-rated health status were positively associated with HGS, while age, having underweight, low cognitive functioning, and functional disability were negatively associated with HGS. In addition among men, higher education and medium economic background were positively and having two or more chronic conditions, having severe depressive symptoms, and having moderate sleep impairment were negatively associated with HGS. Moreover among women, urban residence was negatively associated with HGS (see Table 2).

## 5. Discussion

The study aimed to investigate the patterns and correlates of HGS among older adults (50 years and older) in Indonesia. The mean HGS found in this study among men was 28.2 kgs and among women 17.2 kgs, which was similar to older adults (50 years and older) in India (mean HGS of 28.2 kgs among men and 18.5 kgs among women) [7] and among 60 years and older Singaporeans (28.3 kgs among men and 17.2 kgs among women) [2]. However, the found HGS was lower than in older adults (50 years and older) in China (mean HGS of 34.3 kgs among men and 21.9 kgs in women) [11] and among older adults (50 years and older, mean age 62.0 years) in South Africa (the mean maximum HGS was 37.9 kgs for men and 31.5 kgs for women) [3]. In a study among older adults in 11 European countries, the mean maximum HGS was 41.3 kgs for men and 24.9 kgs for women [25] and among older Japanese-American men mean maximum HGS was reported as 36.7kgs [10]. This finding seems to confirm the lower HGS in developing compared with developed world regions [26], which may be largely explained by differences in body height and body weight [26, 27].

As expected from previous studies [4, 5, 7, 28, 29], HGS was higher in men than in women and decreased with age. The decrease of HGS with age was larger among men (with coefficients ranging from -3.22 to -7.84) than among women (with coefficients ranging from -1.81 to -5.21). This finding that men's HGS level on average decreases faster with age than women's has been found in a number of previous studies [30–32]. Reason for the age and sex differences have been previously well described in terms of degenerative changes and reduction of muscle mass with ageing and men having a larger number of muscle fibres than women [33–35]. In partial agreement with previous studies [7, 24, 36], this study found that among men higher education was associated with higher HGS. This relationship may have been affected by a higher proportion of well-educated men than well-educated women in the relatively small subgroup of well-educated respondents (515 persons). This study found in unadjusted linear regression analysis that urban residence was among men and women associated with higher HGS, while in adjusted linear regression analysis this was no longer significant for men and negatively significant for women. Other studies seem also not to have found clear results regarding urban-rural differences in terms of HGS [e.g., [3]]. While other studies, for example, in India [7], found regional differences in relation to HGS, this study did not find such differences by comparing rates of HGS in Sumatra, Java, and other major island groups.

The anthropometric traits of low height and underweight were found to be associated with low HGS, which is consistent with findings from previous studies [3–5], while having obesity was associated with high HGS. This result was also found in a few studies [6, 37], while other studies [2, 36] found a negative relationship between central obesity and HGS, and other studies found no relationship [4]. Keevill et al. [38] note that “BMI may not be the most appropriate marker of obesity in this context since it incorporates lean mass in its calculation, a determinant of muscle strength” and suggest a better marker would be central obesity. However, in an adjusted model (analysis not shown) central obesity was also highly associated among both men (B=1.92, 95% CI= 1,38, 2.47) and women (B=1.40, 95% CI= 1.05, 1.75) with HGS in this study. It may need to be considered that as some evidence suggests this older age population has higher optimal BMI and waist circumference values than younger people [4, 37]. In bivariate analysis physical activity was among both men and women associated with higher HGS, while in the multivariable model this relationship was no longer significant. A previous study [4] found that higher sitting time and lower practice of physical activity were associated with low HGS, while this study only found in bivariate analysis an association between physical inactivity and low HGS.

As found in a number of previous studies [3, 5, 10, 29, 39–42], this study found among both men and women an association between functional disability and lower HGS and among men a negative relationship between multimorbidity and HGS. It is not clear in this study about the direction of the relationship between functional disability and low HGS, as some studies [e.g., [39]] found low HGS impacting on functional disability. Longitudinal studies are needed to clarify the direction of this relationship.

In agreement with previous studies [3–5, 7–10], this study found that better overall self-rated health and better cognitive functioning were associated with higher HGS. The relationship between better self-rated health and higher HGS could be explained by the fact that self-rated health includes a wide range of information (number of diseases, illness symptoms) [9]. In a review of studies, Fritz et al. [43] found that poorer cognition was associated with weaker HGS. One possible reason for this may be that “motor skill learning and motor output are dependent on the activity of the frontal and parietal brain regions and the interconnection between these regions are related to motor output” [43–45]. In addition, among men in the adjusted model and among women in the bivariate model, poorer mental health status, including severe depressive symptoms, moderate sleep disturbance, and moderate sleep impairment, were associated with lower HGS. These findings seem to confirm results from previous studies [10–12]. It is possible that poor mental health impacts physiological changes such as the metabolic system that in turn may increase lower HGS [11, 46, 47].

To main physical function in an ageing population in Indonesia is relevant for the process of health ageing and activities to improve HGS through for example good nutrition and physical activity are vital [7]. Several modifiable risk factors, such as underweight, having chronic conditions, poor mental health, and cognitive functioning, have been identified that can be targeted in reducing these risk factors and increasing HGS. The subgroup level intervention may target men with lower education and poor mental health.

## 6. Limitations of the Study

This analysis was based on cross-sectional data; therefore, we cannot ascribe causality to any of the associated study variables. However, longitudinal analysis of the IFLS is planned. Data were collected from older adults who were available in the household on the day of the survey, which means participants that were institutionalized (prison, hospital, and care home) were excluded.

## 7. Conclusion

The study found in a large national sample of older adults in Indonesia that the mean HGS was similar to countries in the region such as India and Singapore. Further, the current study identified sociodemographic (age, sex, and educational status), anthropometric (higher, underweight, and overweight/obesity) health (self-rated health, cognitive functioning, functional disability, multiple chronic conditions, and mental health), and correlates of HGS.

## Figures and Tables

**Table 1:** Sample characteristics and mean hand grip strength (HGS) among older adult men and women in Indonesia.

Variable	Sample	Men	Women
		(n=3318, 48.8%)	(n=3779, 51.2%)
*Socio-demographics*		Mean HGS (kg)	

		M (SD)	M (SD)

*All*	7097	28.2 (7.4)	17.2 (5.4)
*Age in yrs (M=61.2, SD=9.4)*			
50-59	3740 (54.8)	30.9 (6.7)	19.0 (4.9)
60-69	2056 (28.8)	26.8 (6.2)	16.6 (4.9)
70-79	1011 (12.7)	22.2 (6.3)	13.3 (4.8)
80+	290 (3.7)	17.9 (6.5)	11.2 (4.8)

Education			
None	1101 (15.7)	24.3 (7.5)	15.1 (5.5)
Elementary	3899 (56.4)	27.6 (7.3)	17.5 (5.2)
High school	1547 (21.1)	30.1 (6.9)	18.4 (5.0)
Higher education	515 (6.8)	30.4 (7.5)	20.0 (5.0)
Missing	35		

Economic background			
Poor	1999 (31.2)	27.6 (6.9)	17.1 (5.3)
Medium	2706 (42.1)	29.3 (7.1)	17.9 (5.1)
Rich	1713 (26.7)	29.0 (7.3)	18.1 (5.2)
Missing	679		

Residential status			
Rural	3212 (48.9)	27.4 (7.6)	17.0 (5.6)
Urban	3885 (51.1)	28.9 (7.2)	17.5 (5.1)

*Region*			
Sumatra	1464 (20.6)	28.4 (7.1)	17.8 (5.0)
Java	4125 (58.1)	28.1 (7.5)	17.1 (5.5)
Major island groups	1508 (21.2)	27.9 (7.2)	17.2 (4.7)

*Anthropometric measures*			

Body Mass Index (BMI)			
Normal	2699 (38.9)	27.6 (7.1)	16.4 (5.0)
Underweight	973 (13.7)	23.5 (6.6)	14.1 (5.1)
Overweight or obesity	3390 (47.4)	30.7 (7.1)	18.5 (5.2)
Missing	35		

*Health variables*			

Cognitive functioning (low)			
No	3754 (70.8)	29.8 (6.9)	18.9 (4.9)
Yes	1552 (29.2)	27.1 (6.8)	16.7 (5.1)
Missing	1791		

Self-rated health status			
Unhealthy	2355 (30.5)	26.4 (7.6)	16.5 (5.2)
Somewhat healthy	3706 (53.7)	28.6 (7.3)	17.5 (5.4)
Very healthy	1036 (15.8)	29.9 (7.1)	17.8 (5.5)

*Functional disability *			
None	5031 (72.2)	29.1 (7.1)	18.0 (5.2)
One	1553 (21.7)	26.1 (7.7)	15.6 (5.1)
Two or more	511 (6.1)	23.9 (7.6)	13.6 (5.9)
Missing	2		

Chronic conditions			
None	3659 (52.6)	28.4 (7.5)	17.1 (5.2)
One	1952 (27.5)	28.1 (7.2)	17.5 (5.7)
Two or more	1485 (19.9)	27.3 (7.5)	17.2 (5.3)
Missing	1		

Physical activity			
Low or inactive	2931 (43.9)	28.4 (7.3)	17.2 (5.3)
Moderate or high	3486 (56.1)	28.9 (7.3)	18.1 (5.1)
Missing	680		

Severe depressive symptoms			
No	6944 (84.5)	28.8 (7.1)	17.7 (5.2)
Yes	372 (5.5)	26.6 (6.2)	16.6 (5.1)
Missing	681		

Moderate sleep disturbance			
No	5433 (85.6)	28.8 (7.2)	17.8 (5.2)
Yes	982 (14.4)	28.1 (6.8)	17.2 (5.4)
Missing	682		

Moderate sleep impairment			
No	5480 (86.0)	28.9 (7.1)	17.8 (5.2)
Yes	925 (14.0)	27.2 (7.2)	16.8 (5.0)
Missing	682		

**Table 2:** Association of hand grip strength with socio-demographic and health variables among older adults in Indonesia by sex.

**Variables**	**Men**		**Women**	
***Socio-demographics***	Unadjusted coefficient	Adjusted coefficient	Unadjusted coefficient	Adjusted coefficient
	estimates: Beta (95% CI)	estimates: Beta (95% CI)	estimates: Beta (95% CI)	estimates: Beta (95% CI)

*Age*				
50-59	Reference	Reference	Reference	Reference
60-69	-4.11 (-4.51 to -3.71)*∗∗∗*	-3.22 (-3.78 to -2.66)*∗∗∗*	-2.41 (-2.70 to -2.12)*∗∗∗*	-1.81 (-2.25 to -1.35)*∗∗∗*
70-79	-8.62 (-9.18 to -8.06)*∗∗∗*	-6.19 (-7.04 to -5.34)*∗∗∗*	-5.74 (-6.12 to -5.31)*∗∗∗*	-3.69 (-4.36 to -3.03)*∗∗∗*
80+	-12.94 (-13.85 to -12.02)*∗∗∗*	-7.84 (-10.02 to -5.65)*∗∗∗*	-7.76 (-8.45 to -7.08)*∗∗∗*	-5.21 (-6.69 to -3.73)*∗∗∗*

Education				
None	Reference	Reference	Reference	Reference
Elementary	3.30 (2.61 to 3.99)*∗∗∗*	1.73 (0.83 to 2.62)*∗∗∗*	2.42 (2.08 to 2.76)*∗∗∗*	-0.24 (-0.75 to 0.27)
High school	5.77 (5.03 to 6.51)*∗∗∗*	1.88 (0.92 to 2.83)*∗∗∗*	3.27 (2.83 to 3.72)	-0.38 (-0.97 to 0.22)
Higher education	6.07 (5.16 to 6.98)*∗∗∗*	1.46 (0.38 to 2.54)*∗∗*	4.85 (4.17 to 5.53)	0.14 (-0.63 to 0.91)

Economic background				
Poor	Reference	Reference	Reference	Reference
Medium	1.70 (1.24 to 2.16)*∗∗∗*	0.62 (0.01 to 1.22)*∗*	0.81 (0.47 to 1.15)*∗∗∗*	0.19 (-0.29 to 0.67)
Rich	1.34 (0.80 to 1.87)*∗∗∗*	0.40 (-0.32 to 1.13)	1.00 (0.64 to 1.37)*∗∗∗*	0.09 (-0.44 to 0.62)

*Residential status*				
Rural	Reference	Reference	Reference	Reference
Urban	1.59 (1.20 to 1.99)*∗∗∗*	0.37 (-0.18 to 0.92)	0.52 (0.24 to 0.80)*∗∗∗*	-0.69 (-1.13 to -0.24)*∗∗*

*Region*				
Sumatra	Reference	---	Reference	Reference
Java	-0.31 (-0.87 to 0.26)		-0.65 (-1.06 to -0.24)*∗∗*	0.05 (-0.44 to 0.54)
Major island groups	-0.51 (-1.33 to 0.32)		-0.58 (-1.16 to 0.01)	0.41 (-0.14 to 0.99)

***Health variables***				

Height (cms)	0.51 (0.48 to 0.54)*∗∗∗*	0.34 (0.30 to 0.38)*∗∗∗*	0.33 (0.31 to 0.35)*∗∗∗*	0.21 (0.17 to 0.26)*∗∗∗*

BMI				
Normal	Reference	Reference	Reference	Reference
Underweight	-4.15 (-4.72 to -3.59)*∗∗∗*	-2.43 (-3.22 to -1.64)*∗∗∗*	-2.29 (-2.72 to -1.83)*∗∗∗*	-1.70 (-2.43 to -0.99)*∗∗∗*
Overweight or obesity	3.07 (2.66 to 3.48)*∗∗∗*	2.02 (1.44 to 2.61)*∗∗∗*	3.07 (2.60 to 3.48)*∗∗∗*	1.29 (0.86 to 1.73)*∗∗∗*

*Health variables*				

Cognitive functioning (low)	-2.67 (-3.13 to -2.21)*∗∗∗*	-1.21 (-1.79 to -0.62)*∗∗∗*	-2.19 (-2.53 to -1.86)*∗∗∗*	-1.35 (-1.82 to -0.89)*∗∗∗*

Self-rated health status				
Unhealthy	Reference	Reference	Reference	Reference
Somewhat healthy	2.18 (1.73 to 2.63)*∗∗∗*	0.45 (-0.18 to 1.08)	1.06 (0.75 to 1.37)*∗∗∗*	0.53 (0.17 to 0.85)*∗∗*
Very healthy	3.48 (1.89 to 4.08)*∗∗∗*	1.23 (0.40 to 2.07)*∗∗*	1.34 (0.90 to 1.78)*∗∗∗*	0.60 (0.13 to 1.06)*∗*

*Functional disability *				
None	Reference	Reference	Reference	Reference
One	-2.99 (-3.49 to -2.53)*∗∗∗*	-0.51 (-1.14 to 0.14)	-2.43 (-2.77 to -2.09)*∗∗∗*	-0.95 (-1.33 to -0.57)*∗∗∗*
Two or more	-5.23 (-6.10 to -4.35)*∗∗∗*	-2.28 (-3.51 to -1.04)*∗∗∗*	-4.38 (-4.92 to -3.84)*∗∗∗*	-1.49 (-2.22 to -0.75)*∗∗∗*

Chronic conditions				
None	Reference	Reference	Reference	Reference
One	-0.35 (-0.82 to 0.13)	-0.24 (-0.86 to 0.38)	0.37 (0.04 to 0.70)*∗*	0.23 (-0.24 to 0.70)
Two or more	-1.16 (-1.71 to -0.60)*∗∗∗*	-1.12 (-1.64 to -0.61)*∗∗∗*	0.16 (-0.20 to 0.51)	-0.004 (-0.51 to 0.50)

Severe depressive symptoms	-2.20 (-3.10 to 1.30)*∗∗∗*	-1.55 (-2.65 to -0.45)*∗∗*	-1.11 (-1.71 to -0.51)*∗∗∗*	-0.18 (-1.07 to 0.71)

Moderate sleep disturbance	-0.65 (-1.25 to -0.06)*∗*	0.47 (-0.24 to 1.20)	-0.54 (-0.93 to -0.15)*∗∗*	-0.04 (-0.51 to 0.51)

Moderate sleep impairment	-1.76 (-2.35 to -1.16)*∗∗∗*	-0.75 (-1.32 to -0.18)*∗∗*	-0.99 (-1.38 to -0.60)*∗∗∗*	-0.31 (-0.86 tp 0.24)

Low physical activity	-0.57 (-0.98 to -0.17)*∗∗*	-0.40 (-0.92 to 0.13)	-0.93 (-1.22 -0.65)*∗∗∗*	-0.40 (-0.79 to 0.002)

*∗∗∗*P<.001; *∗∗*P<.01; *∗*P<.05

## Data Availability

The data for the current study from the Indonesian Family Life Survey (IFLS) are in the public domain and are accessible via the Rand Labor and Population website (https://www.rand.org/labor/FLS/IFLS.html).

## Abbreviations

ADL: Activities of daily living
BMI: Body mass index
CES-D: Centres for Epidemiologic Studies Depression Scale
EA: Enumeration area
HGS: Hand grip strength
IADL: Instrumental activities of daily living
IFLS: Indonesian Family Life Survey
IPAQ: International Physical Activity Questionnaire
PROMIS: Patient-Reported Outcomes Measurement Information System
TICS: Telephone Survey of Cognitive Status.
